# Isolation of Immunocomplexes from Zebrafish Brain

**DOI:** 10.21769/BioProtoc.4646

**Published:** 2023-04-05

**Authors:** Jennifer Carlisle Michel, Adam C. Miller

**Affiliations:** Institute of Neuroscience, University of Oregon, Eugene, OR USA

**Keywords:** Immunoprecipitation (IP), Co-immunoprecipitation (co-IP), Protein complex purification, Whole tissue, Zebrafish, Brain

## Abstract

Zebrafish is an excellent model to study vertebrate neurobiology, but its synaptic components that mediate and regulate fast electrical synaptic transmission are largely unidentified. Here, we describe methods to solubilize and immunoprecipitate adult zebrafish brain homogenate under conditions to preserve electrical synapse protein complexes. The methods presented are well-suited to probe electrical synapse immunocomplexes, and potentially other brain-derived immunocomplexes, for candidate interactors from zebrafish brain.

## Background

**Figure 1.**
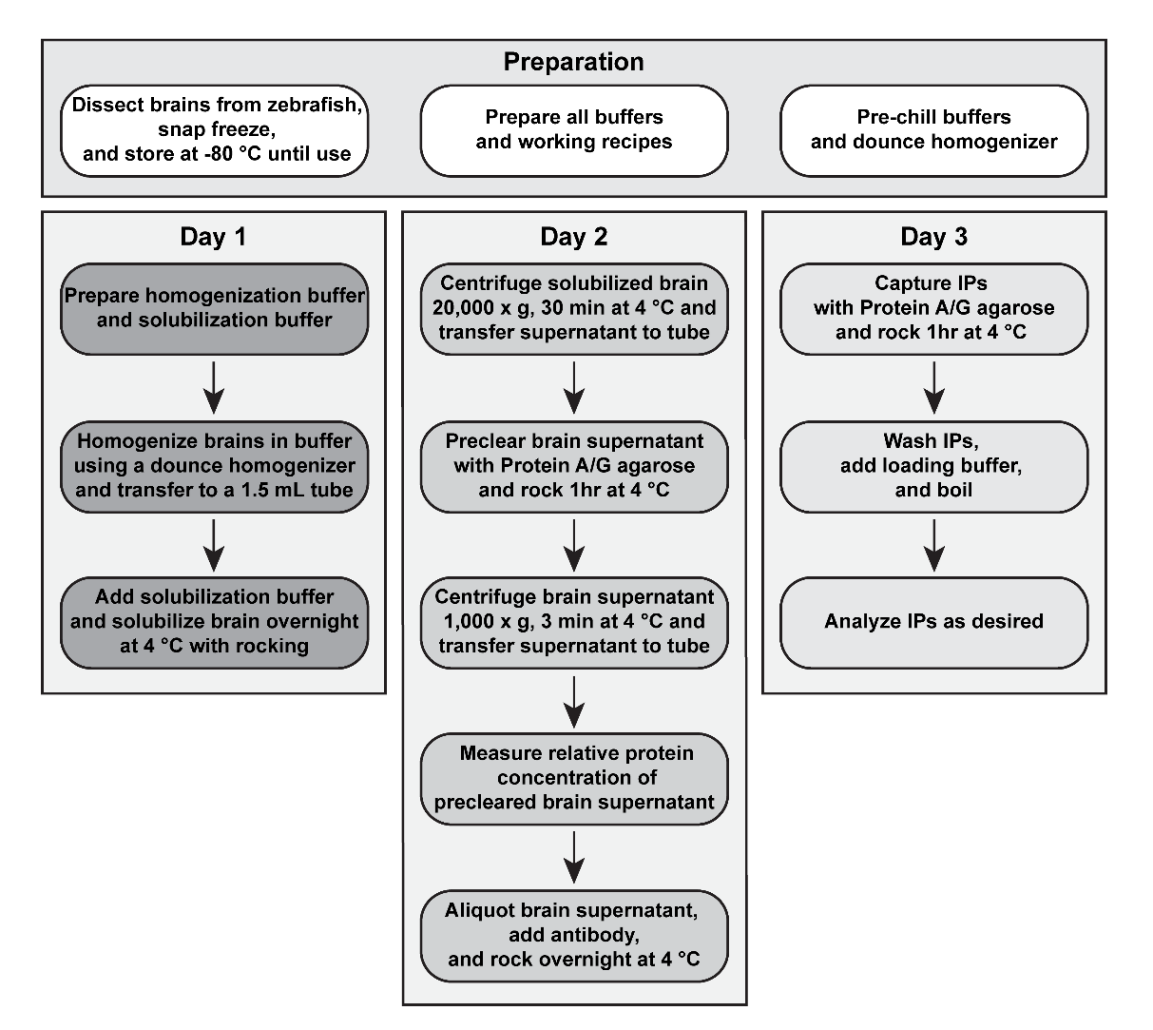
Flowchart for isolation of immunocomplexes from zebrafish brain

Immunoprecipitation (IP) and, under favorable conditions, co-immunoprecipitation (co-IP) can be used to isolate proteins and associated complexes in vivo (Harlow and Lane, 1999). Unlike column affinity chromatography that requires large amounts of starting material, the immunoprecipitation technique enriches target proteins and possibly their associated complexes from a small amount of material. The challenge of successfully isolating immunocomplexes from in vivo tissue is identifying an antibody that specifically and reproducibly binds the target protein, while also co-precipitating directly and indirectly interacting proteins. While polyclonal and monoclonal antibodies each have their own benefits, with the former recognizing multiple epitopes of a target and the latter recognizing a single epitope, it is not possible to predict success a priori. Therefore, it is crucial to test a variety of antibodies to find one that reproducibly immunoprecipitates and co-immunoprecipitates associated complexes. Additional steps critical to successful isolation of immunocomplexes include identifying a detergent that solubilizes the tissue for optimal release of the target protein and maintaining cold temperatures throughout the isolation to prevent protein degradation. Here, we present a protocol optimized to detect electrical synapse immunocomplexes from zebrafish brain (Lasseigne et al., 2021; see flow chart in Figure 1). The method is useful to identify protein–protein interactions in vivo and, though not validated for other tissues, could be adapted to other zebrafish organs and protein complexes, making this a helpful biochemical tool for the zebrafish community.

## Materials and Reagents

1. 1.5 mL plastic cuvettes (Fisher, catalog number: 14-955-127)
2. 1.5 mL Eppendorf Snap-Cap Safe-Lock microcentrifuge tubes (Fisher, catalog number: 05-402-25)
3. 0.65 mL graduated microtube (Fisher, catalog number: NC9260727)
4. Zebrafish brains (from fish ages 3 months–1.5 years) snap frozen in liquid nitrogen and stored at -80 °C
5. Antibody [This will vary with each experiment. We have successfully used anti-ZO1 monoclonal antibody (ZO1-1A12) (ThermoFisher, catalog number: 33-9100) for co-immunoprecipitation.]
6. n-Octyl-β-D-Glucopyranoside (Anatrace, catalog number: O311 5 GM)
7. Pierce protease inhibitor mini tablets, EDTA-free (Fisher, catalog number: PIA32955)
8. Dithiothreitol (DTT) reducing agent (Bio-Rad, catalog number: 610611)
9. HEPES (Fisher, catalog number BP310-500)
10. NaCl (Fisher, catalog number: BP358-212)
11. EDTA, disodium salt dihydrate (Fisher, catalog number: BP120-1)
12. EGTA (Sigma, catalog number: E-4378)
13. NaOH pellets (Fisher, catalog number: M1064621000)
14. Pierce Protein A/G agarose (Fisher, catalog number: PI20421)
15. 4× Laemmli sample buffer (Bio-Rad, catalog number: 1610747)
16. Bio-Rad Protein Assay dye reagent concentrate (Bio-Rad, catalog number: 5000006)
17. 1 M HEPES pH 7.5 (see Recipes)
18. 5 M NaCl (see Recipes)
19. 0.5 M EDTA pH 8.0 (see Recipes)
20. 0.5 M EGTA pH 8.0 (see Recipes)
21. 1 M DTT (see Recipes)
22. Homogenization buffer (see Recipes)
23. Solubilization buffer (see Recipes)
24. Preclear buffer (see Recipes)
25. IP wash buffer (see Recipes)
26. 2× loading buffer (see Recipes)
*Note: See Recipes to create working solutions.*

## Equipment

1. 1 mL dounce tissue grinder with loose and tight pestles (Fisher, catalog number: 50-365-300 or similar)
2. Benchtop tube rocker (Benchmark Scientific M2100 or similar)
3. Refrigerated centrifuge (Eppendorf 5430 R or similar)
4. Spectrophotometer (Eppendorf BioPhotometer or similar)

## Procedure

- **Tissue preparation—Freeze and store brains**
*Note: Adult fish should be euthanized according to proper guidelines for the ethical care and use of animals in research associated with IACUC protocols of the user’s institution.*
  1. Euthanize an adult fish and dissect the brain out as described in Gupta and Mullins (2010; see detailed video at: https://www.jove.com/t/1717).
  2. Transfer each dissected brain to an Eppendorf tube, immediately close the cap tightly, and snap freeze tissue by immersing the tube in liquid nitrogen for at least one minute.
*Note: Liquid nitrogen handling should follow appropriate lab safety guidelines.*
  3. When all brains are dissected, transfer snap frozen brains to -80 °C freezer until use.
*Note: Individual brains are stored separately and combined below dependent upon experimental needs. Frozen brains can be stored at -80 °C for at least two years.*
- **DAY ONE—Homogenize brains**
*Note: The following protocol describes conditions for two IPs. It is important to maintain all reagents on ice throughout the procedure to prevent protein degradation.*
  1. Freshly prepare 5 mL of homogenization buffer by adding one protease inhibitor mini tablet and 5 µL of 1 M DTT; keep on ice.
*Note: Homogenization buffer is prepared in bulk and is sufficient for 50 brains. Use within 60 min of preparation to ensure optimal protease inhibitor activity and DTT stability during tissue homogenization.*
  2. Freshly prepare 5 mL of solubilization buffer by adding 5 µL of 1 M DTT; keep on ice.
*Note: Solubilization buffer is prepared in bulk and is sufficient for 50 brains. Use within 60 min of preparation to ensure DTT stability during tissue solubilization.*
  3. Put five frozen zebrafish brains into an ice-cold 1 mL dounce tissue grinder.
*Note: The resulting quantity of total protein from each brain will vary depending on the age and size of the adult fish dissected. As a general guideline, using two brains per IP often results in 1–2 mg total protein/IP, though the total quantity required will vary according to protein expression levels and antibody efficiency.*
  4. Add 0.5 mL of prepared ice-cold homogenization buffer.
*Note: Save unused homogenization buffer at 4 °C for use on DAY TWO, step C10 to mix the blank solution needed for the protein assay.*
  5. Homogenize brain tissue with 10–20 strokes using the loose pestle and then 10–20 strokes using the tight pestle.
*Note: Homogenizing in the absence of detergent prevents oxidation of proteins. Also, if needed, glass homogenizers and pestles can be decontaminated between samples by rinsing extensively with distilled water, then 70% ethanol, and then distilled water again before drying with air.*
  6. Transfer the homogenate (0.5 mL) to a 1.5 mL Eppendorf tube using a 1,000 µL pipette tip.
  7. Add 0.5 mL of prepared solubilization buffer and gently invert to thoroughly mix the solutions.
*Note: The prepared solubilization buffer contains 4% detergent, so that when mixed with an equal volume of brain homogenate, the final detergent concentration is 2%. Save unused solubilization buffer at 4 °C for use on DAY TWO, step C10 to mix the blank solution needed for the protein assay.*
  8. Solubilize the brain tissue by rocking the tube on a benchtop rocker at 24 rpm, overnight at 4 °C.
- **DAY TWO—Preclear and set up immunoprecipitations**
  1. Prepare pre-washed Pierce Protein A/G agarose by washing a 250 µL bed volume with 1 mL of preclear buffer.
*Note: Pre-washed Pierce Protein A/G agarose is prepared in bulk and is sufficient for three preparations.*
  2. Spin at 1,000 *× g* for 3 min at 4 °C.
  3. Remove buffer and repeat wash steps C1 and C2 two more times using new preclear buffer each time.
  4. After the final wash, remove buffer and resuspend agarose in 250 µL of preclear buffer to achieve a 500 µL 50% slurry of pre-washed Pierce Protein A/G agarose. Store pre-washed agarose at 4 °C until use in step C7 below and in DAY THREE, step D2.
  5. Next, centrifuge the overnight solubilized brain homogenate at 20,000 *× g* for 30 min at 4 °C.
  6. Without disturbing the pellet, transfer the solubilized brain supernatant to a new 1.5 mL Eppendorf tube using a 1,000 µL pipette tip.
  7. Preclear the brain supernatant by adding 100 µL of 50% slurry of pre-washed Pierce Protein A/G agarose (prepared in steps C1–C4), and rock on benchtop rocker at 24 rpm for 1 h at 4 °C.
  8. Spin the supernatant at 1,000 *× g* for 3 min at 4 °C to pellet the agarose.
  9. Without disturbing the agarose, carefully transfer the precleared supernatant to a new 1.5 mL Eppendorf tube using a 1,000 µL pipette tip.
  10. Measure the relative quantity of total protein using Bio-Rad protein assay according to the manufacturer’s instructions (see detailed protocol at https://www.bio-rad.com/webroot/web/pdf/lsr/literature/LIT33.pdf; 1.5 mL plastic cuvettes and a spectrophotometer are required).
*Note: For the blank solution, mix equal quantities of homogenization buffer and solubilization buffer that were saved at 4 °C from DAY ONE, steps B4 and B7.*
  11. Save an extract sample by transferring 40 µL of precleared brain supernatant to a new 0.65 mL microtube and adding 40 µL of 2× loading buffer. Save at -20 °C until needed for SDS-PAGE electrophoresis on DAY THREE, step D12.
  12. To set up IPs, transfer 400 µL of precleared brain supernatant to each of two new 1.5 mL Eppendorf tubes (this is approximately 1–2 mg total protein/IP).
*Note: If comparing IPs from different samples, be sure to immunoprecipitate an equal quantity of total protein in an equal volume. The minimum volume for an IP is 200 µL. Also, use the precleared extract for immunoprecipitation immediately. Do not freeze the precleared supernatant or the protein complexes will fall apart, and co-IP of associated proteins will fail.*
  13. Add 2 µg of antibody per IP.
*Note: Anywhere from 0.5–5 µg can be used, depending on the efficiency of the antibody.*
  14. Rock the IPs on a benchtop rocker at 24 rpm, overnight at 4 °C.
- **DAY THREE—Capture immunoprecipitates**
  1. Centrifuge the IPs at 1,000 *× g* for 1 min at 4 °C.
  2. Add 30 µL of 50% slurry of pre-washed Pierce Protein A/G agarose (prepared in DAY TWO steps C1–C4 and stored at 4 °C) to each IP.
  3. Rock IPs on a benchtop rocker at 24 rpm for 1 h at 4 °C.
  4. Centrifuge IPs at 1,000 *× g* for 3 min at 4 °C.
  5. Remove supernatant with a 1,000 µL pipette tip without disturbing the agarose.
  6. Add 1 mL of IP wash buffer to the agarose and invert several times.
  7. Spin 1,000 *× g* for 3 min at 4 °C.
  8. Repeat wash steps D5–D7 two more times, using new IP wash buffer each time.
  9. On the last wash, completely remove the wash buffer from the agarose using a 200 µL pipette tip, followed by a flattened 10 µL gel loading tip.
  10. Add 20 µL of 2× loading buffer.
  11. Boil extract sample and immunoprecipitations for 3 min at 95 °C.
    *Note: Boiling may cause aggregation of membrane proteins. In this case, alternative options include omitting step D11 or heating the samples at 65* °C *for 10 min instead. Best results need to be empirically determined per experiment.*
  12. Continue with protein analysis as desired, e.g., SDS-PAGE and western blot (Towbin et al., 1979; Sambrook and Russell, 2006; Ni et al., 2017).
    *Note: The subsequent protein analysis steps are dependent upon the equipment available. For a comprehensive guide, see* *https://www.bio-rad.com/webroot/web/pdf/lsr/literature/Bulletin_6040.pdf*.

## Recipes

1. **1 M HEPES, pH 7.5 (500 mL)**
  Dissolve 119.15 g of HEPES (MW 238.5 g/mol) in 400 mL of ultrapure water. Adjust pH to 7.5 with 10 N NaOH. Adjust final volume to 500 mL with ultrapure water.
2. **5 M NaCl (500 mL)**
  Dissolve 146 g of NaCl (MW 58.44) in 400 mL of ultrapure water. Adjust final volume to 500 mL with ultrapure water.
3. **0.5 M EDTA, pH 8.0 (100 mL)**
  Dissolve 18.61 g of EDTA, disodium salt dihydrate (MW 372.24 g/mol) in 80 mL of ultrapure water. Adjust the pH to 8.0 with NaOH pellets. Adjust the final volume to 100 mL with ultrapure water.
4. **0.5 M EGTA, pH 8.0 (100 mL)**
  Dissolve 19.02 g of EGTA (MW 380.35 g/mol) in 80 mL of ultrapure water. Adjust the pH to 8.0 with 4 N NaOH. Adjust final volume to 100 mL with ultrapure water.
5. **1 M DTT (10 mL)**
  Dissolve 1.5 g of DTT in 8 mL of ultrapure water. Adjust volume to 10 mL, dispense into 1 mL aliquots, and store at -20 °C.
6. **Homogenization buffer (50 mL)**
  20 mM HEPES pH 7.5 1.0 mL 1 M HEPES pH 7.5
  150 mM NaCl 1.5 mL 5 M NaCl
  5 mM EDTA 0.5 mL 0.5 M EDTA
  5 mM EGTA 0.5 mL 0.5 M EGTA
   46.5 mL ultrapure water
7. **Solubilization buffer (25 mL)**
  20 mM HEPES pH 7.5 0.5 mL 1 M HEPES pH 7.5
  150 mM NaCl 0.75 mL 5 M NaCl
  5 mM EDTA 0.25 mL 0.5 M EDTA
  5 mM EGTA 0.25 mL 0.5 M EGTA
  4% n-Octyl-β-D-Glucopyranoside  1 g n-Octyl-β-D-Glucopyranoside
   Adjust final volume to 25 mL with ultrapure water
8. **Preclear buffer (10 mL)**
  20 mM HEPES pH 7.5 5 mL homogenization buffer
  150 mM NaCl 5 mL solubilization buffer
  5 mM EDTA 
  5 mM EGTA 
  2% n-Octyl-β-D-Glucopyranoside
9. **IP wash buffer (25 mL)**
  20 mM HEPES pH 7.5 23.75 mL homogenization buffer
  150 mM NaCl 1.25 mL solubilization buffer
  5 mM EDTA
  5 mM EGTA
  0.2% n-Octyl-β-D-Glucopyranoside
10. **2× loading buffer (1 mL)**
  2× Laemmli sample buffer 0.5 mL 4× Laemmli sample buffer
  200 mM DTT 0.2 mL 1 M DTT
   0.3 mL ultrapure water
